# Performance of Prognostic Scoring Systems in MINOCA: A Comparison among GRACE, TIMI, HEART, and ACEF Scores

**DOI:** 10.3390/jcm12175687

**Published:** 2023-08-31

**Authors:** Damiano Fedele, Lisa Canton, Francesca Bodega, Nicole Suma, Francesco Pio Tattilo, Andrea Impellizzeri, Sara Amicone, Ornella Di Iuorio, Khrystyna Ryabenko, Matteo Armillotta, Angelo Sansonetti, Andrea Stefanizzi, Daniele Cavallo, Marcello Casuso, Davide Bertolini, Luigi Lovato, Emanuele Gallinoro, Marta Belmonte, Andrea Rinaldi, Francesco Angeli, Gianni Casella, Alberto Foà, Luca Bergamaschi, Pasquale Paolisso, Carmine Pizzi

**Affiliations:** 1 Cardiology Unit, Cardiac Thoracic and Vascular Department, IRCCS Azienda Ospedaliera-Universitaria di Bologna, 40138 Bologna, Italylucabergamaschi91@gmail.com (L.B.); 2 Department of Medical and Surgical Sciences—DIMEC, Alma Mater Studiorum, University of Bologna, 40138 Bologna, Italy; 3 Pediatric and Adult CardioThoracic and Vascular, Onchoematologic, and Emergency Radiology Unit, IRCSS Azienda Ospedaliera-Universitaria di Bologna, 40138 Bologna, Italy; 4 Clinical Cardiology and Cardiovascular Imaging Unit, Galeazzi-Sant’Ambrogio Hospital, IRCCS, 20157 Milan, Italy; 5 Department of Biomedical and Clinical Sciences, University of Milan, 20157 Milan, Italy; 6 Department of Advanced Biomedical Sciences, University of Naples Federico II, 80138 Naples, Italy; martabelmonte@coreaalst.com; 7 Cardiovascular Center Aalst, OLV Hospital, 9300 Aalst, Belgium; 8 Unit of Cardiology, Maggiore Hospital, 40131 Bologna, Italy

**Keywords:** MINOCA, prognosis, GRACE score, TIMI score, HEART score, ACEF score

## Abstract

**Background**: the prognosis of patients with myocardial infarction with non-obstructive coronary arteries (MINOCA) is not benign; thus, prompting the need to validate prognostic scoring systems for this population. **Aim**: to evaluate and compare the prognostic performance of GRACE, TIMI, HEART, and ACEF scores in MINOCA patients. **Methods**: A total of 250 MINOCA patients from January 2017 to September 2021 were included. For each patient, the four scores at admission were retrospectively calculated. The primary outcome was a composite of all-cause death and acute myocardial infarction (AMI) at 1-year follow-up. The ability to predict 1-year all-cause death was also tested. **Results**: Overall, the tested scores presented a sub-optimal performance in predicting the composite major adverse event in MINOCA patients, showing an AUC ranging between 0.7 and 0.8. Among them, the GRACE score appeared to be the best in predicting all-cause death, reaching high specificity with low sensitivity. The best cut-off identified for the GRACE score was 171, higher compared to the cut-off of 140 generally applied to identify high-risk patients with obstructive AMI. When the scores were tested for prediction of 1-year all-cause death, the GRACE and the ACEF score showed very good accuracy (AUC = 0.932 and 0.828, respectively). **Conclusion:** the prognostic scoring tools, validated in AMI cohorts, could be useful even in MINOCA patients, although their performance appeared sub-optimal, prompting the need for risk assessment tools specific to MINOCA patients.

## 1. Introduction

Myocardial infarction with non-obstructive coronary arteries (MINOCA) is a heterogeneous clinical condition, representing 6–11% of the total population with AMI [1]. It is more prevalent in young, non-white, and female patients [2,3,4,5]. Since MINOCA prognosis is not as benign as traditionally assumed, the need to develop and validate prognostic scoring systems to stratify individual risk has emerged [4,5,6].

In acute coronary syndromes (ACSs), the most widespread tool to predict high-risk patients with worse outcomes is the Global Registry of Acute Coronary Events (GRACE) score [7]. Being initially conceived to identify patients at high risk of in-hospital mortality, it was then validated to predict the risk of all-cause death at long-term follow-up, up to 5 years [8,9,10,11,12,13]. Similarly, the Thrombolysis in Myocardial Infarction (TIMI) score is a scoring tool developed in non-STE-segment elevation ACS (NSTE-ACS) patients and validated in predicting 5-year all-cause mortality [14,15]. The HEART score was developed specifically for risk stratification in chest pain patients with suspected NSTE-ACS at the emergency department, and then it was shown to also be accurate when predicting worse outcomes at long-term follow-up [15,16,17,18,19]. Conversely, the ACEF score was originally proposed and validated to assess mortality risk in elective cardiac surgery, being then applied to also predict outcomes in patients with ACSs [20,21,22]. However, the accuracy of the mentioned prognostic scores in predicting outcomes in a population of solely MINOCA patients and a direct comparison among them remains to be evaluated [15].

Thus, our study aimed to evaluate and compare the prognostic performance of GRACE, TIMI NSTE-ACS, HEART, and ACEF scores in MINOCA patients.

## 2. Materials and Methods

### 2.1. Study Population

In this multicenter, observational study, all patients from the AMIPE Registry (“Acute Myocardial Infarction, Prognostic, and Therapeutic Evaluation”; ClinicalTrials.gov number: NCT03883711) admitted with AMI at S. Orsola-Malpighi and Maggiore Hospitals, from January 2017 to September 2021, were screened. Patients with unknown coronary anatomy, incomplete clinical data to assess all the scores, and missing follow-up data were excluded. Diagnosis of STEMI and NSTEMI, referral, and timing of invasive coronary angiography (ICA) were managed according to the current European Society of Cardiology (ESC) Guidelines [1,23,24]. The diagnosis of MINOCA was made according to the ESC Guidelines [1,25]. Other specific causes of acute myocardial injury were ruled out through cardiac magnetic resonance, pulmonary, and/or vascular computed tomography when clinically indicated. GRACE, TIMI NSTE-ACS, HEART, and ACEF scores at admission were retrospectively calculated according to the validated criteria [7,14,19,20]. The variables included in each score are shown in Table 1.

### 2.2. Study Endpoints

The primary endpoint of the study was to evaluate and compare the prognostic performance of GRACE, TIMI NSTE-ACS, HEART, and ACEF scores in MINOCA patients. The primary outcome was a composite of all-cause death and AMI [“major adverse event” (MAE)] at 1-year follow-up. In addition, the ability to predict 1-year all-cause death was also tested.

### 2.3. Statistical Analysis

The normality distribution of continuous variables was assessed visually using histograms and Q-Q plots. Continuous variables with normal distribution were expressed as the mean ± standard deviation and non-normally distributed variables as the median [interquartile range]. Categorical variables were expressed as counts and percentages. Differences between groups were analyzed using the t-test and the Mann–Whitney U-test for continuous variables, when appropriate. The χ^2^ test or Fisher’s Exact test was used to compare group differences, as appropriate. The performance of prognostic scores in predicting long-term outcomes was assessed with the area under the curve (AUC) of the receiver operating characteristic (ROC) curves. Comparison among AUC was tested with the DeLong test. The Youden index was used to identify the best cut-off of each score for the prediction of the primary endpoint. The population was divided according to these cut-offs, and survival was estimated with Kaplan–Meier curves and compared using the log-rank test. The correlation among scores was assessed with the Spearman rank test. All analyses were performed using the Statistical Package for Social Sciences version 28.0 (SPSS, PC version, Chicago, IL, USA) and Stata version SE 18.0 (Stata Corp LLC, College Station, TX, USA). Statistical significance was defined as two-tailed *p*-values < 0.05.

## 3. Results

**Figure 1:**
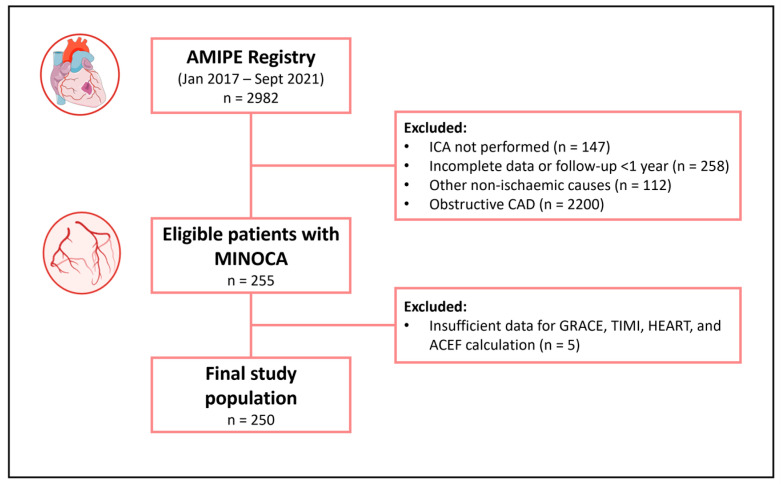
Flowchart of the study. Abbreviations: AMIPE = Acute Myocardial Infarction, Prognostic, and Therapeutic Evaluation; CAD = coronary artery disease; ICA = invasive coronary angiography; MINOCA = myocardial infarction with non-obstructive coronary arteries.

The study population eligible for the study consisted of 255 consecutive MINOCA patients with at least 1-year follow-up available. Five patients were excluded for insufficient data for prognostic risk score calculation. Thus, the final study population included 250 patients with MINOCA. Overall, the mean age was 68 [53–78] years, and 163 (65.2%) were females. Only one patient died (0.4%) during the index hospitalization, while three (1.2%) patients had a recurrent AMI within 30 days of follow-up. At the 1-year follow-up, 11 patients (4.4%) had died, of which 6/11 (54%) were cardiovascular deaths, and 8 (3.2%) presented a recurrent AMI. Accordingly, patients were divided into two groups: patients who experienced MAEs (composite endpoint of all-cause death and AMI) at the 1-year follow-up (n = 19, 7.6%) and patients who did not (n = 231). The flowchart of the study is shown in Figure 1.

### 3.1. Admission Clinical Features Included in the Prognostic Scores

Clinical, laboratory, and instrumental characteristics at admission are presented in Table 2. Compared to patients who did not experience MAE, patients who experienced it were older (*p* < 0.001), more frequently females (*p* = 0.021), with a higher prevalence of type-2 diabetes mellitus (*p* = 0.034), arterial hypertension (*p* = 0.029), and chronic obstructive pulmonary disease (*p* = 0.040). Patients reporting the composite endpoint presented lower systolic blood pressure (*p* = 0.008), a higher rate of atrial fibrillation (AF) (*p* = 0.008), with overall higher Killip class (*p* = 0.029) at admission compared to patients with better prognosis. Finally, patients presenting MAE had higher levels of inflammatory markers and lower levels of hemoglobin compared to patients who did not experience the composite endpoint (*p* < 0.05 for all). Therapy at discharge included dual antiplatelet therapy in 113 patients (45.2%), beta-blockers in 187 (74.8%), inhibitors of the renin–angiotensin–aldosterone system in 158 (63.2%), and statins in 185 patients (74%).

### 3.2. Prognostic Scoring Systems’ Performance

**Figure 2:**
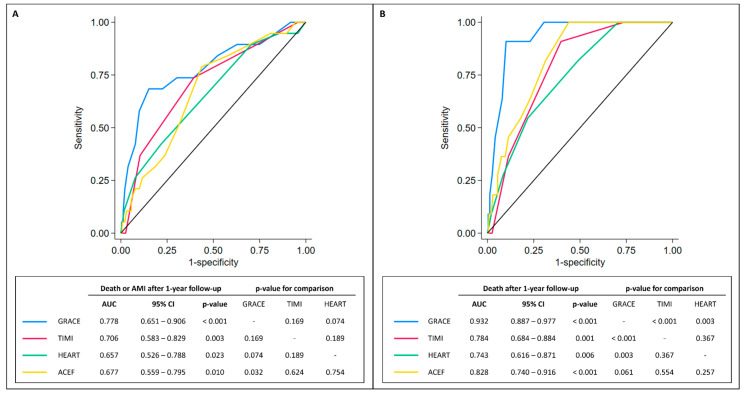
ROC curves. Panel (**A**). ROC curves comparing GRACE, TIMI, HEART, and ACEF scores in predicting long-term all-cause death or AMI (after 1 year). Panel (**B**). ROC curves comparing GRACE, TIMI, HEART, and ACEF scores in predicting long-term all-cause death. Abbreviations: 95% CI = 95% confidence interval; AMI = acute myocardial infarction; AUC = area under the curve.

In our cohort of MINOCA, the median scores were 128 [105–157] for the GRACE, 2 [1–3] for the TIMI NSTE-ACS, 7 [5–7] for the HEART, and 1.11 [0.90–1.34] for the ACEF. ROC curves of GRACE, TIMI NSTE-ACS, HEART, and ACEF scores in predicting 1-year outcomes are displayed in Figure 2.

Interestingly, all the presented scores showed sub-optimal performance in predicting MAE in MINOCA patients with an AUC < 0.8. Among them, the GRACE score appeared to be superior to ACEF (*p* for comparison = 0.032) in predicting MAE. When the scores were tested for 1-year all-cause death, only the GRACE and the ACEF scores showed very good performance (>0.80) in predicting a worse prognosis (AUC = 0.932, 95% CI 0.887–0.977 for GRACE; AUC = 0.828, 95% CI 0.740–0.961 for ACEF). Similarly, the GRACE score was superior to the HEART and TIMI NSTE-ACS scores in predicting the 1-year all-cause death (*p* = 0.003 and *p* < 0.001, respectively). Of note, no other significant differences among the other scores were noted, with only a moderate correlation (0.3 < r < 0.7) among them (Table 3).

**Figure 3:**
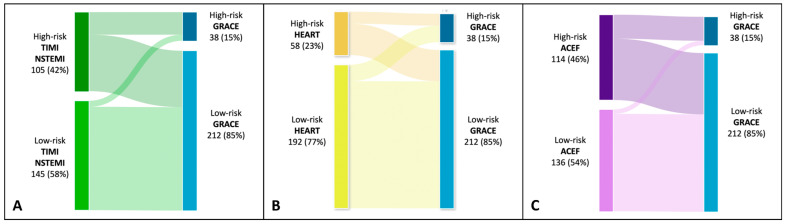
Alluvial plot showing mutual relationships among risk class identified by GRACE, TIMI, HEART, and ACEF scores. Panel (**A**). High-risk class by TIMI if >2; high-risk class by GRACE if >171. Panel (**B**). High-risk class by HEART if >5. Panel (**C**). High-risk class by ACEF if >1.16.

As shown in the alluvial plot (Figure 3), TIMI, HEART, and ACEF scores included in the high-risk category a considerable proportion of patients that the GRACE score categorizes as low-risk. The HEART score is the one in which a larger proportion of patients included in the low-risk category turn out to be at high-risk with the GRACE score. In contrast, for both TIMI and ACEF, only a small proportion of patients defined as low-risk turn out to be at high-risk for the GRACE score.

### 3.3. Best Cut-Offs and Survival Analyses

Using the Youden index, the best cut-offs of each score to predict 1-year all-cause death and AMI prediction were identified. The accuracy indicators of each score are reported in Table 4.

**Figure 4:**
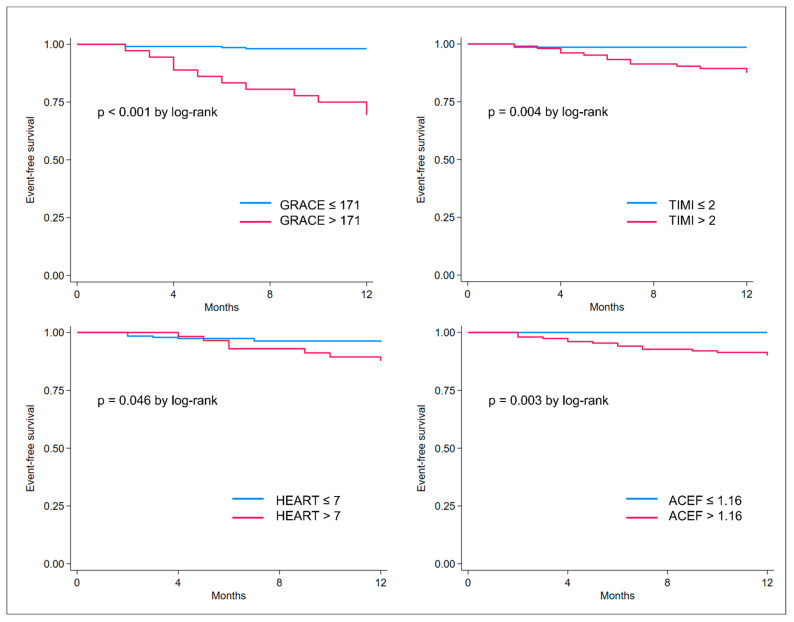
Kaplan–Meier curves showing event-free survival (death or acute myocardial infarction) in risk categories as defined per the best cut-off identified for GRACE, TIMI, HEART, and ACEF scores.

The cut-offs for each score were the following: 171 for the GRACE score, 2 for the TIMI NSTE-ACS, 1.16 for the ACEF, and 7 for the HEART score. Interestingly, the best cut-off identified for the GRACE score was 171, which is higher compared to the cut-off of 140 generally applied for the identification of high-risk patients with obstructive NSTE-ACS. These cut-offs were used for dichotomizing the study population into high and low risk for each prognostic score; thus, calculating the survival curves presented in Figure 4. For all the scores, high-risk patients presented statistically significantly lower survival at 1-year follow-up compared to low-risk patients (log-rank *p* values < 0.001 for the GRACE; *p* = 0.004 for the NSTE-ACS; *p* = 0.046 for the HEART score, and *p* = 0.003 for the ACEF score).

## 4. Discussion

In this study, for the first time, we compared, in a population of solely MINOCA patients, the accuracy of the most commonly used scores predicting prognosis in ACSs. The main findings were as follows: (i) overall, the scores showed sub-optimal performance in predicting the composite MAE in MINOCA patients with an AUC ranging between 0.7 and 0.8; (ii) the GRACE score appeared to be the best prognostic score in predicting all-cause death in MINOCA, reaching high specificity with low sensitivity; (iii) the best cut-off identified for the GRACE score was 171, higher compared to the cut-off of 140 generally applied for the identification of high-risk patients with obstructive NSTE-ACS; (iv) when the scores were tested for 1-year all-cause death, only the GRACE and the ACEF scores showed very good performance (>0.80) in predicting the worse prognosis.

### 4.1. Prognostic Scores in MINOCA

To the best of our knowledge, this is the first study comparing the most used prognostic scores in a population of solely MINOCA patients. So far, only the GRACE score and the ACEF score have been tested in MINOCA [29,30]; however, a direct comparison of the prognostic accuracy of the available scores in the same MINOCA population has not been reported yet. In our study, the GRACE and ACEF scores showed a very good performance in predicting 1-year all-cause death. Nonetheless, their AUC in identifying patients at high risk of death and AMI at 1-year follow-up appeared sub-optimal (AUC < 0.80). Indeed, the prognostic performance of these scores in patients with ACSs was reported as very good [15,17,26,27,29].

**Figure 5:**
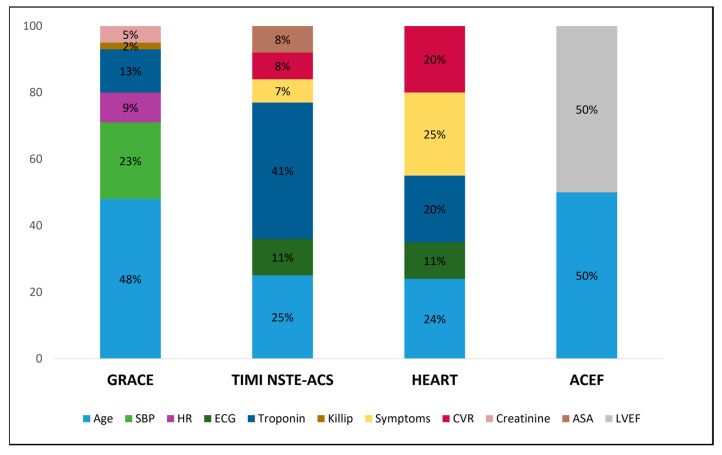
Stacked bar plot showing the relative mean weight of each item in determining the maximum GRACE, TIMI, HEART, and ACEF score per patient. Of note, the weight of items included in the ACEF score was estimated to be 50% for either age or LVEF since it was calculated as age/LVEF (+1 if serum creatinine >2 mg/dL) and only a small percentage of patients had serum creatinine >2 mg/dL. Abbreviations: ASA = acetylsalicylic acid; CVR = cardiovascular risk factors; HR = heart rate; LVEF = left ventricle ejection fraction; NSTE-ACS = non-ST-elevation acute coronary syndrome; SBP = systolic blood pressure.

These findings might be explained by the following considerations: (i) the prognostic scores were originally designed to stratify the patients’ risk at short-term and then validated at long-term follow-up; in our study, we only evaluated the composite endpoint at the l-year follow-up, due to the relatively low event rate at 30 days from the index hospitalization; (ii) the GRACE score itself was originally designed to predict the risk of mortality and then proven to accurately predict mortality and AMI at the 1-year follow-up; (iii) the heterogeneity of MINOCA patients, presenting different etiopathology of the acute event, with some sub-groups being at higher risk (e.g., young females [31]); (iv) these scores were tested mainly in patients with obstructive AMI (due to the high prevalence of obstructive AMI in the scenario of ACSs), who have a different cardiovascular profile compared to patients with MINOCA. Among all the scores tested, the GRACE score was the best in predicting 1-year all-cause death in the MINOCA population. This is probably due to the high “weight” attributed to the “Age” variable, which also represents one of the strongest independent predictors of all-cause death in MINOCA patients [31,32] (Figure 5).

### 4.2. Risk Categories in MINOCA

In the present study, the best cut-offs to predict all-cause death and re-AMI at the 1-year follow-up were identified for all the prognostic scores tested. Interestingly, the best cut-off for the GRACE score in our cohort was 171, higher compared to the cut-off of 140 generally applied to identify high-risk patients with obstructive NSTE-ACS [33]. Similarly, Yin et al. reported the cut-off of 159 for the GRACE score to predict a composite of cardiac death, non-fatal AMI, stroke, heart failure, and cardiovascular rehospitalization in the MINOCA population [34]. This higher cut-off might reflect the overall better prognosis of MINOCA patients compared to MIOCA, with a lower but not negligible risk of cardiovascular adverse events at long-term follow-up [3,4,35,36,37]. This appears to be even more relevant, considering the overall lower GRACE score reported in MINOCA patients compared to MIOCA ones [38]. A GRACE score >171 reached high specificity (89%) with low sensitivity (68%). Among the scores tested in our study, the ACEF score with a cut-off of >1.16 showed the highest sensitivity (79%). The latter was already tested in MINOCA patients; however, a lower cut-off (1.02) was proposed to identify high-risk patients [30]. Applying the same cut-off in our cohort enhanced sensitivity to 90%, but PPV was 11%, and accuracy was 44%. This discrepancy might be due to the longer follow-up period (3.5 years) with more adverse events considered by Gao et al. in their study [30]. Overall, the GRACE score seems to perform better than the other tested scores, with a higher accuracy in predicting all-cause death compared to the composite outcome. Thus, our results confirm that the applicability of this score is feasible also in MINOCA patients.

### 4.3. Clinical Implications

The identification of patients with high-risk of adverse events at short- and long-term follow-up might be helpful in guiding a rational resource allocation. Our study confirmed that in patients with lower-risk scores, adverse events are particularly rare. Nonetheless, our data showed that MINOCA patients labeled as “high risk” by prognostic scoring systems could experience MAE (all-cause death and AMI) even at 1-year follow-up. Thus, these risk assessment tools might be applied to select patients who might benefit the most from (i) early invasive management (as also proved by the GRACE score in obstructive AMI), including the use of intravascular imaging and/or functional tests in the MINOCA setting [39,40,41,42]; (ii) more “aggressive”/adequate optimization of the medical therapy with an antiplatelet agent, statins, and renin–angiotensin–aldosterone system inhibitors, which seem to be effective also in patients with MINOCA [5,43,44,45,46]; and (iii) a closer follow-up after discharge.

However, it is crucial to highlight that none of the presented scores showed very good performance, with AUC > 0.80 in predicting MAE in MINOCA patients. Thus, a new and more targeted prognostic scoring system, including variables shown to be independent predictors of adverse outcomes in MINOCA patients, should be developed, including (i) clinical features (e.g., female sex, diabetes mellitus, ST-deviation at ECG) [47,48,49,50] and (ii) instrumental findings (presence of fibrosis at late gadolinium enhancement at cardiac magnetic resonance) or high-risk plaque features identified at intravascular imaging [51,52,53,54,55,56,57,58,59,60].

### 4.4. Limitations of the Study

Our study has some limitations. First, the relatively small sample size and low incidence of MAE might affect the statistical power. Nevertheless, our population of MINOCA patients was cautiously selected with the exclusion of possible non-cardiac causes of myocardial injury, myocarditis, cardiomyopathies, or takotsubo syndrome. Furthermore, the pathological mechanism of MINOCA was not systematically evaluated with intracoronary imaging and functional coronary tests. For these reasons, our findings should be interpreted as hypothesis-generating and further prospective and adequately powered studies are needed to confirm our findings.

## 5. Conclusions

Since the rate of MAEs in MINOCA patients is not negligible, prognostic scoring systems, validated in wide AMI cohorts, could also be useful in MINOCA patients. In particular, the GRACE score showed the best performance, although the cut-off to identify high-risk patients was higher compared to one used in patients with ACSs. Overall, the performance of these prognostic scores was sub-optimal in the setting of MINOCA, prompting the need for tailored risk-stratification tools for MINOCA patients.

## Figures and Tables

**Table 1:** Summary of GRACE, TIMI NSTE-ACS, HEART, and ACEF scores.

SCORE	GRACE	TIMI NSTE-ACS	HEART	ACEF
**Original tested outcome**	In-hospital mortality [7]	14-day mortality, recurrent AMI, or urgent revascularization [14]	Long-term mortality, AMI, PCI/CABG [19]	Peri-operative mortality in elective cardiac surgery [20]
**Further tested outcomes**	▪ 1-year mortality or AMI * [26] ▪ Up to 5-year mortality [15]	▪ 30-days death, type 1 AMI, PCI/CABG * [27] ▪ 1 to 5-year mortality [15]	▪ 30-days death, type 1 AMI, PCI/CABG * [27] ▪ Up to 6-moths death, AMI, PCI/CABG * [17,28] ▪ 2-years death or AMI * [18] ▪ Up to 5-years mortality [15]	▪ 30-days mortality after PCI in AMI [21] ▪ 1-year mortality after PCI in AMI [21,22] ▪ 1-year death, AMI, PCI/CABG, stent thrombosis, TIA/ictus [22]
**ITEMS (in brackets the minimum and maximum scoring, or score if present)**				
**Age**	▪ Age (0–100)	▪ Age ≥65 yy (1)	▪ Age <45 yy (0) ▪ Age 45–64 yy (1) ▪ Age ≥65 yy (2)	Age/LVEF (+1 if creatinine >2 mg/dL)
**Past medical history**	-	▪ ≥3 CVR (HBP, DM, HC, family history, smoke history) (1) ▪ Known CAD (1) ▪ ASA <7 dd (1)	▪ No CVR (0), ▪ 1–2 CVR (HBP, DM, HC, obesity, smoke history, family history) (1) ▪ ≥3 CVR or Atherosclerosis (2)	
**Vital signs**	▪ SBP (0–58) ▪ HR (0–46)	-	-	
**Clinical presentation**	▪ Killip class (0–59) ▪ Cardiac arrest (0–39)	▪ ≥2 episodes of angina <24 h (1)	▪ History: ▪ low clinical suspect (0) ▪ moderate clinical suspect (1) ▪ high clinical suspect (2)	
**ECG**	▪ ST deviation (0–28)	▪ ST deviation (1)	▪ Normal (0), ▪ ST deviation not due to LBBB/LVH/digoxin (2) ▪ other repolarization abnormalities (1)	
**Laboratory test**	▪ Abnormal cardiac enzymes (0–14) ▪ Creatinine (0–28)	▪ Abnormal cardiac enzymes (0–1)	▪ cTn < URL (0) ▪ cTn 1x-URL-3x (1) ▪ cTn > 3x URL (2)	
**RISK CLASS**				
**Low risk**	<109 in NSTE-ACS <126 in STEMI	0–2	0–3	<1.0 in PCI in AMI [21] ≤1.45 in ACS [22]
**Intermediate risk**	109–140 in NSTE-ACS 126–154 in STEMI	3–4	4–6	1–1.39 in PCI in AMI [21] 1.451–2.0 in ACS [22]
**High risk**	>140 in NSTE-ACS >154 in STEMI	5–7	7–10	≥1.4 in PCI in AMI [21] >2.0 in ACS [22]

* In these validation studies, using high-sensitivity cardiac troponin did not alter the performance of the score. Abbreviations: ACS = acute coronary syndrome, AMI = acute myocardial infarction, ASA = acetylsalicylic acid, CABG = coronary artery bypass grafting, CAD = coronary artery disease, cTn = cardiac troponin, CVR = cardiovascular risk factor, dd = days, DM = diabetes, HBP = high blood pressure, HR = heart rate, LBBB = left bundle branch block, LVEF = left ventricular ejection fraction, LVH = left ventricular hypertrophy, NSTE-ACS = non-ST-segment elevation acute coronary syndrome, PCI = percutaneous coronary intervention, SBP = systolic blood pressure, TIA = transitory ischemic attack, URL = upper reference limit, yy = years.

**Table 2:** Baseline characteristics and comorbidities stratified by the occurrence of major adverse events (the composite endpoint) at 1 year of follow-up.

	Total N = 250	MAE N = 19	No MAE N = 231	*p*-Value
**Age, years**	68 [53–78]	78 [69–87]	67 [53–77]	<0.001
**Female, n (%)**	163 (65.2)	17 (89.5)	146 (63.2)	0.021
**BMI, kg/m^2^**	25.7 [22.8–28.8]	25.9 [23.7–28.1]	25.6 [22.7–28.8]	0.694
**Cardiovascular risk factors**				
**Current smoking, n (%)**	46 (18.4)	1 (5.3)	45 (19.5)	0.214
**Hypertension, n (%)**	167 (66.8)	17 (89.5)	150 (64.9)	0.029
**Dyslipidemia, n (%)**	162 (64.8)	11 (57.9)	151 (65.4)	0.618
**Type-2 diabetes, n (%)**	26 (10.4)	5 (26.3)	21 (9.1)	0.034
**Family history of CVD, n (%)**	45 (18.0)	2 (10.5)	43 (18.6)	0.540
**Comorbidities**				
**Previous stroke, n (%)**	16 (6.4)	3 (15.8)	13 (5.6)	0.111
**COPD, n (%)**	27 (10.8)	5 (26.3)	22 (9.5)	0.040
**PAD, n (%)**	6 (2.4)	0 (0)	6 (2.6)	0.477
**Clinical presentation**				
**Typical chest pain ^a^, n (%)**	138 (55.2)	7 (36.8)	131 (56.7)	0.148
**Killip ≥2, n (%)**	25 (10.0)	5 (26.3)	20 (8.7)	0.029
**SBP, mmHg**	138 ± 27.3	122 ± 31.8	139 ± 26.4	0.008
**HR, bpm**	80 [69–98]	90 [74–120]	80 [68–96]	0.089
**STEMI, n (%)**	28 (11.2)	3 (15.8)	25 (10.8)	0.455
**AF, n (%)**	26 (10.4)	6 (31.6)	20 (8.7)	0.008
**WBC, cell/nL**	8.6 [6.9–11.1]	9.9 [7.4–13.1]	8.5 [6.9–11.0]	0.040
**Hb, g/dL**	13.4 ± 1.87	12.5 ± 1.84	13.5 ± 1.90	0.034
**Glucose, mg/dL**	107 [96–129]	111 [100–158]	107 [95–129]	0.127
**Creatinine, mg/dL**	0.8 [0.7–1.0]	0.9 [0.7–1.0]	0.8 [0.7–1.0]	0.750
**LDL cholesterol, mg/dL**	119 ± 37.4	104 ± 25.3	120 ± 37.3	0.093
**Troponin (1st sample), ng/L**	78 [40–330]	132 [52–804]	77 [40–327]	0.149
**LVEF, %**	58 ± 8.9	58 ± 8.6	57 ± 11.7	0.999
**Risk scores**				
**GRACE**	128 [105–157]	179 [132–204]	126 [104–151]	<0.001
**TIMI NSTE-ACS**	2 [1–3]	3 [2–4]	2 [1–3]	0.002
**HEART**	7 [5–7]	7 [6–9]	6 [5–7]	0.020
**ACEF**	1.11 [0.90–1.34]	1.26 [1.17–1.64]	1.10 [0.90–1.33]	0.010

Continuous variables are presented as mean ± SD or median [LQ-UQ], when indicated; categorical ones as n (%). Abbreviations: AF = atrial fibrillation; AMI = acute myocardial infarction; BMI = body mass index; COPD = chronic obstructive pulmonary disease; CVD = cardiovascular disease; Hb = hemoglobin; HR = heart rate; LVEF = left ventricle ejection fraction; MAE = major adverse event; PAD = peripheral artery disease; SBP = systolic blood pressure; STEMI = ST-elevation myocardial infarction; WBC = white blood cells. ^a^ = Typical chest pain was defined retrosternal sensation of pain, pressure, or heaviness (‘angina’) radiating to the left arm, both arms, the right arm, the neck, or the jaw, which may be intermittent (usually lasting several minutes) or persistent [1].

**Table 3:** Correlations among GRACE, TIMI NSTE-ACS, HEART, and ACEF scores.

	GRACE	TIMI NSTE-ACS	HEART
**GRACE**	-	-	-
**TIMI NSTE-ACS**	0.49 *	-	-
**HEART**	0.42 *	0.68 *	-
**ACEF**	0.67 *	0.46 *	0.36 *

* Statistically significant (*p* < 0.001).

**Table 4:** Cut-offs and diagnostic performance of scores.

	Sensitivity	Specificity	PPV	NPV	Accuracy
**GRACE > 171**	68%	89%	34%	97%	88%
**TIMI NSTE-ACS > 2**	74%	61%	13%	97%	62%
**HEART > 7**	42%	78%	14%	94%	76%
**ACEF > 1.16**	79%	57%	13%	97%	58%

Abbreviations: NPV = negative predictive value, PPV = positive predictive value.

## Data Availability

The datasets used and/or analyzed during the current study are available from the corresponding author upon reasonable request.
